# Simultaneous Determination of Seven Phenolic Acids, Two Flavonoids, and Tussilagone in Rat Plasma after Administration of *Farfarae Flos* Extract by LC–MS/MS

**DOI:** 10.1155/2022/1942900

**Published:** 2022-03-24

**Authors:** Guangying Yu, Dan Fang

**Affiliations:** ^1^Department of Traditional Chinese Medicine, Tianjin Hospital, Tianjin 300211, China; ^2^Heping Hospital of No. 983 Hospital of Joint Service Support Force, Tianjin 300020, China

## Abstract

A simple LC-MS/MS method was established for the simultaneous quantitative analysis of concentration of seven phenolic acids, two flavonoids, and tussilagone in biological samples. The lower limit of quantitation of each target compound was less than 10 ng·mL^−1^. The precision of these three types of compounds was less than 15%, and all accuracy was between 85.9% and 115%. The preliminary pharmacokinetics of these three types of compounds in plasma samples were carried out using LC-MS/MS after administration of *Farfarae Flos* extract (3.90 and 7.80 g·kg^−1^) to rats, respectively. The results showed that *T*_max_ of all ten compounds varied from 0.21 ± 0.04 h to 0.69 ± 0.19 h. Maximum concentrations and area under concentration-time curves of seven analyzed phenolic acids were higher than those of the two flavonoids and tussilagone. Terminal elimination half-life of tussilagone was the shortest among these three types of compounds. The results showed that the developed LC-MS/MS method was suitable for clarifying the pharmacokinetic characteristics of these three types of compounds in plasma after administration of *Farfarae Flos* extract in rats.

## 1. Introduction

*Farfarae Flos* has been used as the herbal medicine to treat the diseases for thousands of years in China. It is the dried flower bud of the perennial herbal plant *Tussilago farfara* L and is named as Kuandonghua in China [1]. *Farfarae Flos* has been used as a main herbal medicine to relieve bronchitic and asthmatic conditions in clinic. It has many pharmacological effects including antioxidant [2], antimicrobial [3], antitubercular, [4] and inhibitory a-glucosidase activity [5]. Furthermore, some literature studies have reported that *Farfarae Flos* could be applied to the treatment of pulmonary, obesity, type 2 diabetes, and even hepatitis [3, 5].

The compounds in *Farfarae Flos* include some phenolic acids, flavonoids, terpenoids (tussilagone), and pyrrolizidine alkaloids [6]. These complex compounds have different biological activities. Phenolic acids and flavonoids from *Farfarae Flos* have antimicrobial activity [3]. Tussilagone is selected as an index to evaluate its quality of *Farfarae Flos* in the content determination item in Chinese Pharmacopoeia (2020). It was reported that tussilagone had the anti-inflammatory and antitumor effects [7–9]. In order to clarify the pharmacological effect, it is very important to detect the concentration of these three types of compounds in the biosamples after intragastric administration of *Farfarae Flos*.

At present, there were a great deal of methods to detect the content of active components in the medicinal materials and decoction pieces of *Farfarae Flos* samples. The contents of phenolic acids, senkirkine, and senecionine were quantitatively assayed by HPLC and LC-MS methods, respectively [10, 11]. Meanwhile, GC-MS and NMR spectroscopies were developed and validated to investigate and identify the metabolic fingerprinting of *Farfarae Flos* [12, 13]. However, there is no bioanalysis method to determine these three types of compounds including flavonoids, terpenoids and phenolic acids in the plasma and to study the pharmacokinetics characteristics of three types of compounds after intragastric administration of *Farfarae Flos* extract.

In present study, the concentrations of seven phenolic acids, two flavonoids, and tussilagone in plasma samples were successfully, quantitatively, and simultaneously analyzed by the newly developed LC-MS/MS method. All pharmacokinetic characteristics of three types of compounds successfully fully illuminated after intragastric administration of *Farfarae Flos* to rat.

## 2. Experiments

### 2.1. Chemicals and Reagents

**Figure 1:**
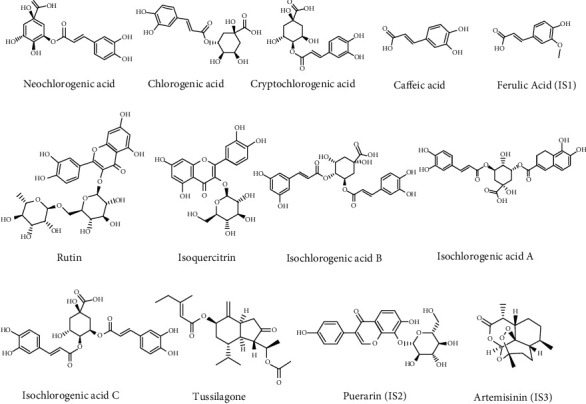
Structures of 13 compounds.

The thirteen reference standards (Figure 1) such as neochlorogenic acid (NCLA), caffeic acid, rutin, isoquercitrin, chlorogenic acid (CLA), tussilagone, puerarin, ferulic acid, cryptochlorogenic acid (CCLA), isochlorogenic acid C (IAC), isochlorogenic acid A (ICA), isochlorogenic acid B (IAB), and artemisinin were obtained from Meilian Biotechnology Company (Sichuan, China). The purity of 13 standards is larger than 98%. Chromatographic grade acetonitrile and methanol are commercially purchased from Merck (Darmstadt, Germany). The deionized water was produced by using a Milli-Q ultrapure water system. Mass spectrum purity grade of reagent formic acid was purchased from Concord Reagent Company (Tianjin, China).

### 2.2. *Farfarae Flos* Sample Preparation

*Farfarae Flos* were purchased from a herbal market of Anguo city (Hebei, China). The plant was identified and authenticated as *Farfarae Flos* by Dr. Guangying Yu (Department of Traditional Chinese Medicine, Tianjin Hospital). The sample of *Farfarae Flos* was storied in Tianjin Hospital (Tianjin, China). One kilogram of the raw *Farfarae Flos* sample was extracted using 10 liters of 95% ethanol for 2 hours by a reflux method, and the supernatant was added into the container. The residue was then extracted by 60% ethanol solution (10 Liters) for another 2 hours. The extract solutions were mixed together and then concentrated at 40°C until they were dry. A final extract of 376 g was obtained and stored at −80°C in a refrigerator.

### 2.3. Preparation of Different Types of Solutions

Stock solution of 10 analytical compounds (1.00 mg·mL^−1^) and three internal standards (ISs) was dissolved in methanol and placed in the refrigerator before testing. Methanol was used to prepare ferulic acid (IS_1_), puerarin (IS_2_), and artemisinin (IS_3_) solutions to reach a concentration of 1 *μ*g mL^−1^, respectively. A specific volume of each analyte stock solution is mixed together to prepare the standard working solution containing 10 analytes with a concentration of 10 times at LLOQ and three levels (high, medium, and low). Quality control (QC) samples for methodological validation were prepared to obtain the final concentration by adding standard working solution (10 *μ*L) into blank plasma samples (100 *μ*L).

Four concentration levels of NCLA, CCLA, and IAC in QC samples was prepared at 2, 6, 200, and 6000 ng·mL^−1^ for LLOQ, low, medium, and high level, respectively. Four concentration levels of CLA, rutin, isoquercitrin, and tussilagone were 1, 3, 100, and 3000 ng·mL^−1^, respectively. Four concentration levels of ICB and ICA were 4, 12, 400, and 12000 ng mL^−1^, respectively. Four concentration levels of caffeic acid were 10, 30, 1000, and 30000 ng·mL^−1^, respectively.

### 2.4. LC-MS/MS Conditions

A high-performance liquid chromatography instrument was used to separate three types of compounds (Agilent 1200 series, USA). The HPLC instrument is composed of four parts; a G1322A degasser, G1312A Bin Pump, G1367B auto-sampler, and G1316A thermostatic column compartment. Three types of compounds and three internal standards (ISs) were separated on an Eclipse Plus C18 (4.6 × 100 mm, 1.8 *μ*m). The mobile phase was composed of formic acid aqueous solution (A) and formic acid methanol solution (B). The concentration of formic acid in both A and B solution was 0.1% (v/v). The gradient elution was as follows. The proportion of 10% B changes to 40% from 0 min to 3mins. 40% B changes to 90% from 16 to 18 min. B is maintained at 100% from 19 to 28 min. The flow rate, sample injected volume, and column temperature was 0.35 mL·min^−1^, 10 *μ*L, and 35°C, respectively.

Quantitative analysis of all samples was performed on Sciex API 3200 with an electrospray ionization (ESI) source. Analyst 1.6.2 workstation for instrument operation, data management, and data analysis were employed to analyze and process data (AB, Sciex). Ten analyzed compounds and three ISs were optimized during multiple reactions monitoring (MRM). The optimized data are shown in Table 1. Briefly, MRM parameters of NCLA were set at 353.2-191.0. MRM parameters of CLA were set at 353.0-191.0. MRM parameters of CCLA were set at 353.1-191.0. MRM parameters of caffeic acid, rutin, isoquercitrin was set at 179.1-135.1, 609.3-300.2, 463.3-300.1, respectively. MRM parameters of IAB were set as 515.3-353.2 and MRM parameters of IAA were set at 515.2-353.0. MRM parameters of IAC and tussilagone were set as 515.3-352.9, 391.4-217.4, respectively.

Phenolic acids (seven acids and IS_1_) and flavonoids (rutin, isoquercitrin, and IS_2_) were detected in the negative ionization mode in the first 23 minutes. Tussilagone and IS_3_ were detected in the positive ion mode in the next 6 minutes by mass spectrometry. The key source parameters in negative ion mode/in positive ion mode were 4500 V/5000 V ion spray voltage, 500°C/350°C temperature, 25 psi/30 psi curtain Gas, 8 psi/8 psi collision Gas, 30 psi/45 psi ion Source Gas 1, 55 psi/55 psi ion Source Gas 2, respectively.

### 2.5. Method Validation

To develop the HPLC-MS/MS method more creditably, some key parameters of method validation were validated based on bioanalytical method validation guidelines of USA Food and Drug Administration [14].

### 2.6. Specificity

Biological blank plasma samples were independently taken from 6 rats. The specificity of the newly established LC-MS/MS was investigated by analyzing the chromatograms of blank biological samples, the simulated biological samples with three mixed reference standards at LLOQ, and the chromatograms of real plasma at 30 minutes after intragastric administration of *Farfarae Flos* extract to exclude endogenous interference. The peak of all analyzed compounds should separate each other. It needs to be found that there were no interferences in blank plasma samples.

### 2.7. Lower Limit of Quantitation, Linearity, Matrix Effect, and Extraction Recovery

The signal to noise ratio of ten analyzed components should be at least more than 5 at LLOQ. The accuracy should range from 80% to 120% of the nominal concentration while RSDs of determined concentration should be less than 20%. Calibration curves of ten analyzed components were prepared by adding different concentrations of reference standards into blank plasma samples. The stock solutions of ten reference standards were continuously diluted with methanol to achieve the linear concentration of the solutions, as follows: NCLA, CCLA, and IAC (20-60000 ng·mL^−1^); caffeic acid (100-300000 ng·mL^−1^); CLA, isoquercitrin, rutin, and tussilagone (10-30000 ng·mL^−1^); IAB and IAA (40-120000 ng·mL^−1^). The ratio of peak area/IS of 10 analytical components to their actual concentration was used to construct the standard curve by weighted least squares when 1/*x*^2^ was selected as weighting factor.

The matrix effects of tested compounds were investigated by measuring the percentage of peak areas of 10 compounds added to the blank rat plasma matrix after extraction and the peak areas in the corresponding working solution at three concentration levels. The extraction recoveries of ten compounds were tested by measuring the percentage of the average peak areas of ten compounds added to blank plasma sample and the average peak areas of ten compounds added to blank matrix after extraction. The matrix effect extract and extraction recoveries at each concentration with RSD <15% were acceptable.

### 2.8. Precision and Accuracy

The precision and accuracy are tested on the same day and three consecutive days and calculated by QC samples with low, medium, and high concentrations, respectively. RSD of the calculated concentration of each compound was calculated at three concentration of QC samples. The intraday and interday accuracy of the determination of each compound was calculated by the percentage of the measured concentration and the spiked concentration. The RSD value of precision needs to be less than 15%. Accuracy is required to be between 85% and 115%.

### 2.9. Stability

In the stability test, 24-hour stability (The samples were placed for 24 hours at 20°C and then measured), freeze-thaw cycles (The sample was injected after the temperature changing from −20°C to 20°C), and 4 weeks stability (The sample was determined after stored at −80°C for 4 weeks) of representative QC samples were all tested. All of them were assayed with six replicates.

### 2.10. Pharmacokinetic Study

Twenty male rats (4 months old, weight 240 ± 20 g) were obtained commercially from Vital River Laboratory Animal Company (Beijing, China). Before the pharmacokinetic experiments, they were free to eat food and drink water for one week for adapting to the environment when the dark light cycle was 12 hours, while the temperature was maintained at 25 ± 5°C. The animal experiments were guided and strictly approved by Tianjin Hospital animal ethics committee. They were fasted for 12 hours and could only drink freely water before intragastric administration of *Farfarae Flos* extract. Ten rats in each group were employed for eliminating data variation caused by different animal individuals. All rats in groups 1 and group 2 were orally administrated one time at two dosages of *Farfarae Flos* extract (carboxymethyl cellulose sodium salt (0.5%) was used to suspend the extract to prepare the administration solution 3.90 g·kg^−1^ and 7.80 g·kg^−1^), respectively. Rats were anesthetized with ether. The heparinized 1.5 mL polythene tube was used to collect bank plasma samples before administering dose of the extract. About 250 *μ*L plasma samples were collected at each collecting points of 5 min, 10 min, 0.25 h, 0.50 h, 0.75 h, 1 h, 2 h, 4 h, 8 h, 12 h, 24 h, and 36 h via fossa orbitalis after dosing. All plasma samples from collecting points need to be centrifuged at 4,000 rpm for ten minutes. The supernatant fluid of centrifuged plasma samples was obtained and immediately stored at −20°C in a refrigerator until they were analyzed.

The software DAS 1.0 (Anhui, China) was adopted to calculate the main pharmacokinetic parameters of 10 target compounds such as area under concentration-time curve (AUC), elimination half-life (*t*_1/2_), and mean residence time (MRT). The maximum concentration (*C*_max_) and the time to reach the maximum concentration (*T*_max_) of ten target compounds were directly calculated by the real concentration-time data in the plasma. The data were expressed as mean ± SD.

### 2.11. Plasma Sample Extraction Method

Ten microliters of ISs solution (contained puerarin, ferulic acid, and artemisinin) and the plasma sample (100 *μ*L) were added into a clean tube, and then acetonitrile (400 *μ*L) was added to precipitate protein. After centrifugation at 14000 rpm for 10 minutes, a mild nitrogen flow was used to evaporate the liquid of the supernatant to dryness. Then, the residue was dissolved in methanol (100 *μ*L). The solution was vortexed for 3 min, mixed for 3 minutes with ultrasound, and centrifuged for 10 min. Finally, the solution (10 *μ*L) was used to detect concentration of ten target compounds in rat plasma.

## 3. Results and Discussion

### 3.1. Internal Standards Selection

The internal standards should have the similar chemical properties and suitable retention time comparable to the analytes. In our experiments, 3 different types of compounds were analyzed. Therefore, ferulic acid was selected as the IS_1_ for phenolic acid. Puerarin was chosen as the IS_2_ for flavonoids. Artemisinin was selected as IS_3_ for tussilagone.

### 3.2. Optimization of LC-MS/MS Conditions

**Figure 2:**
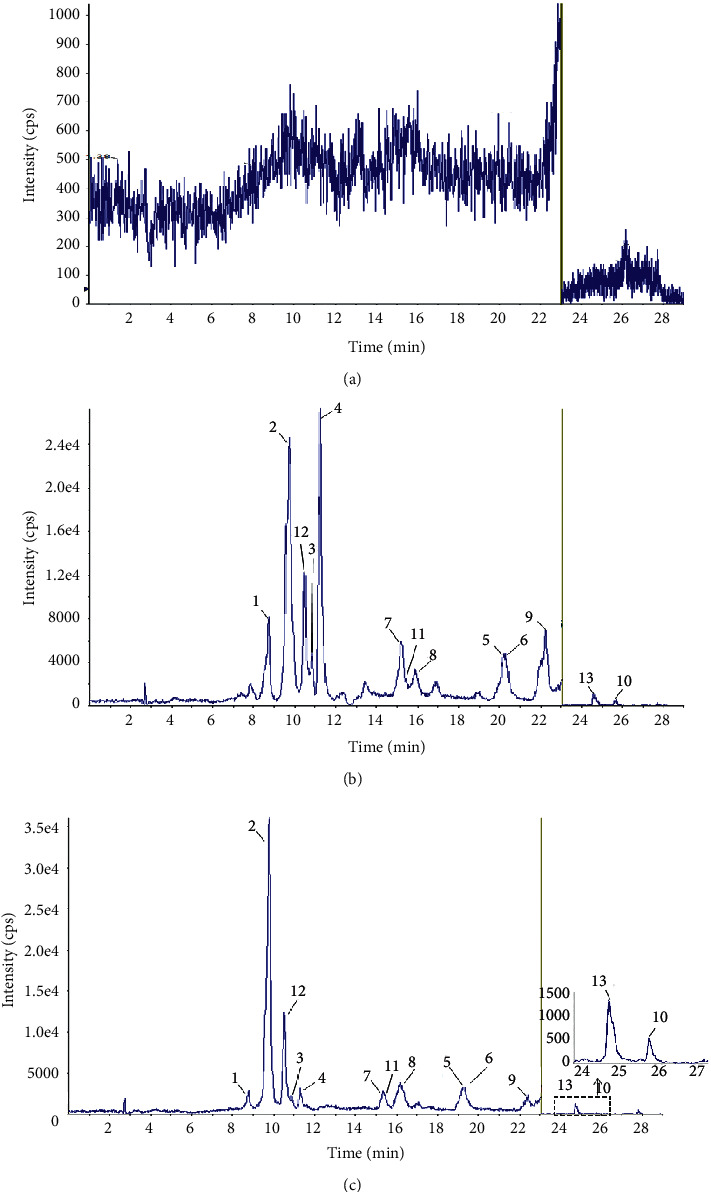
Total LC-MS chromatograms of 13 compounds. (a) blank plasma; (b) blank rat plasma spiked with standard compounds; (c) real sample taken from rats. 1 = NCLA, 2 = CLA, 3 = CCLA, 4 = caffeic acid, 5 = rutin, 6 = isoquercitrin, 7 = IAB, 8 = IAA, 9 = IAC, 10 = tussilagone, 11 = IS_1_, 12 = IS_2_, 13 = IS_3_.

**Figure 3:**
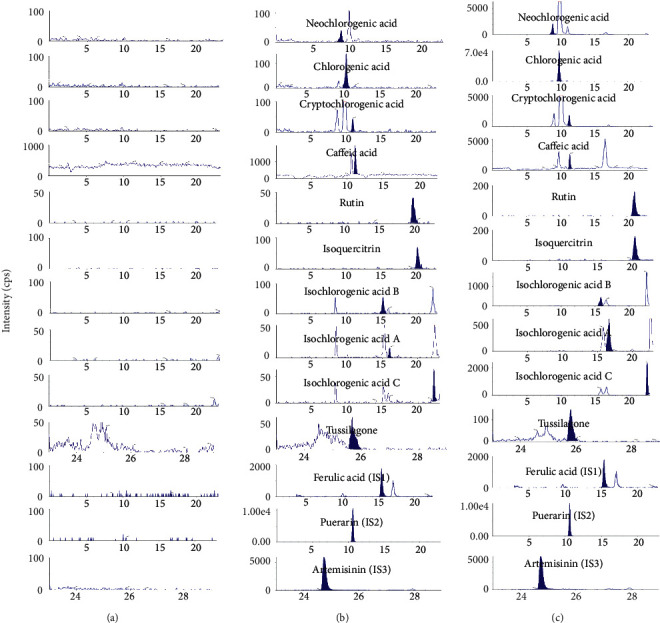
Typical LC-MS/MS chromatograms (a) blank rat plasma, (b) blank plasma spiked with ten analytes and three ISs at LLOQ level, (c) real plasma sample after administration o*f Farfarae Flos*.

The better compounds separation and peak shape could be obtained when the mobile phase was composed of formic acid aqueous solution and formic acid methanol solution. Ten target compounds and the three ISs were separated well within 29 min. For the mass conditions, the ten analyzed compounds were optimized to obtain a great sensitivity of them. In the first 23 minutes, flavonoids and phenolic acids were detected in the negative ion mode. In the next 6 minutes, tussilagone and artemisinin were detected in positive ion mode (Figures 2 and 3). Retention times of NCLA, CLA, CCLA, caffeic acid, rutin, isoquercitrin, IAB, IAA, IAC, and tussilagone were 8.73 min, 9.75 min, 10.80 min, 11.21 min, 20.52 min, 20.77 min, 15.19 min, 15.51 min, 22.45 min, and 25.61 min, respectively. Retention times of IS_1,_ IS_2_, and IS_3_ were 15.43 min 10.48 min, and 24.69 min, respectively.

### 3.3. Method Validation

The LC-MS/MS chromatograms of ten target compounds (phenolic acids, flavonoids, and tussilagone) in rat plasma are shown in Figure 3. It was found that there were no endogenous and exogenous substances interfering with the detection of target compounds in the real plasma samples. All compounds showed good linearities according to the correlation coefficients (*r*^2^ > 0.9917). The LLOQs of ten compounds were less than 10 ng ml^−1^. The detailed information is listed in Table 2.

The detailed precision and accuracy of ten compounds in QC samples at three different concentration and LLOQ levels are shown in Table 3. RSDs of ten compounds in QC sample at LLOQ were lower than 20%. The accuracy of ten compounds at LLOQ ranged from 80.6% to 112%. Meanwhile, the accuracy of ten compounds in QC sample at other three different concentrations ranged from 85.9% to 116%. All RSDs of ten compounds at three different concentration levels were below 14.6%. According to the above results, the new LC-MS/MS method could be acutely to simultaneously detect the concentration of phenolic acids, flavonoids, and tussilagone in rat plasma.

The recovery and matrix effect of these ten compounds and three ISs are listed in Table 4. It was indicated that the mean recoveries of these ten compounds in rat plasma were more than 80.3% and less than 114%. The matrix effects of these ten compounds ranged from 85.1% to 115%. Recoveries of ferulic acid, puerarin, and artemisinin were more than 70%. The matrix effect of three ISs ranged from 91.9% to 95.6% with RSD less than 11%. These data showed that acetonitrile precipitation protein was a reliable method for extracting ten compounds from plasma samples. It was found that ten target compounds were stable in 24 h stability, three freeze-thaw, and four-weeks stability tests (Table 5).

### 3.4. Pharmacokinetics Study

**Figure 4:**
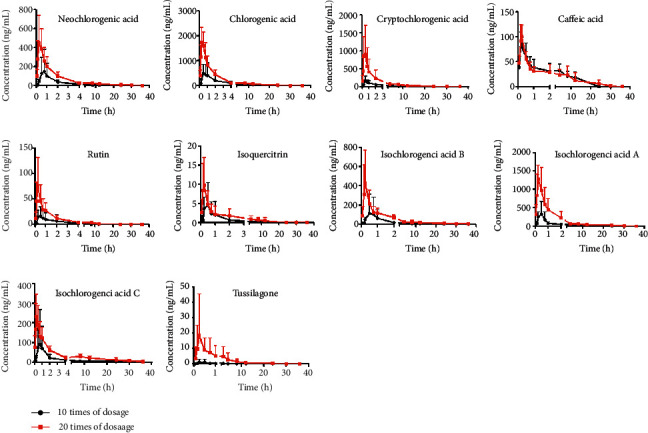
Concentration-time curves of 10 compounds in rats.

The pharmacokinetic analysis of seven phenolic acids, two flavonoids, and tussilagone were successfully performed by HPLC-MS/MS. The compartmental analysis was employed to obtain the main key pharmacokinetic parameters of ten bioactive components. The results showed that the pharmacokinetic behavior of seven phenolic acids, two flavonoids, and tussilagone were more consistent with the one compartment model. The concentration-time curves of seven phenolic acids, two flavonoids, and tussilagone in the plasma are shown in Figure 4. It was found that *C*_max_ of each compound increased with the increase of administration dose (Table 6). *C*_max_ of CLA, IAA, and CCLA was more than 1000 ng·mL^−1^ in the plasma after oral administration of 7.80 g·kg^−1^ extract while *C*_max_ of tussilagone was 18.07 ± 12.99 ng·mL^−1^. This result showed that three phenolic acids (CLA, CCLA, and IAA) possessed higher exposure concentration *in vivo* than other seven components. It suggested that these components should be preferentially screened for active ingredients from *Farfarae Flos* extract to treat some diseases. It was found that *T*_max_ of ten components was less than 0.69 ± 0.19 h. This result showed that ten components was absorbed rapidly after intragastric administration of *Farfarae Flos* extract. *T*_max_ of seven phenolic acids was in the range of 0.21 ± 0.04 to 0.69 ± 0.19 h. The mean residence time (MRT_(0–24h)_) (h) values ranged 4.17 ± 1.73 h to 8.80 ± 2.73 h. In previous pharmacokinetic study on chlorogenic acid [15], *T*_max_ and MRT of chlorogenic acid was 0.70 ± 0.19 h and 5.08 ± 0.89 h, respectively, which is similar to the current research.

Focus on the pharmacokinetic study of flavonoids, *T*_max_ values of rutin and isoquercitrin were closer at low-dose and high-dose group extract. MRT_(0–24h)_ of rutin were 1.95 ± 1.06 h and 2.79 ± 0.24 h after intragastric administration of extract at low-dose and high-dose groups, respectively. The *t*_1/2_ (h) values of rutin were 0.61 ± 0.46 h and 0.82 ± 0.51 h, respectively. The *C*_max_ value of tussilagone was 0.83 ± 0.50 ng·mL^−1^ after intragastric administration of 3.9 g·kg^−1^*Farfarae Flos*, which was closer to the LLOQ of tussilagone. Therefore, studying on the high-dose group (7.8 g·kg^−1^) was necessary. After intragastric administration of 7.8 g·kg^−1^ of *Farfarae Flos* extract, *C*_max_ of tussilagone reached 18.07 ± 12.99 ng·mL^−1^. Tussilagone was absorbed after intragastric administration and reached *T*_max_ within 0.40 ± 0.33 h of tussilagone. The value of *t*_1/2_ (h) was 0.82 ± 0.72 h. These pharmacokinetic parameters will contribute to the clinical application of *Farfarae Flos* extract.

## 4. Conclusion

An accurate, rapid, and reproducible LC-MS/MS method was successfully established to perform the pharmacokinetic analysis of seven phenolic acids, two flavonoids, and tussilagone after intragastric administration of *Farfarae Flos*. The advantage of this method was that simultaneous detection of ten bioactive components in the plasma can be completed in a run with the relative LLOQs. The pharmacokinetic characteristics of these three types of compounds was obtained in rat plasma after intragastric administration of *Farfarae Flos* extract. Limitations of this study were that only preliminary pharmacokinetic studies about three types of compounds (ten bioactive components) have been carried out and more information needs to be in depth explored in the future. The pharmacokinetic profiles of ten active compounds were characterized for the first time, which will be useful for future clinical applications of *Farfarae Flos*.

## Figures and Tables

**Table 1:** MRM parameters of ten compounds and three ISs.

Compound	Parent ion	Daughter ion	Declustering potential (V)	Entrance potential (V)	Collision energy (V)	EXP (V)
NCLA	353.2	191.0	−25	−3	−22	−4.5
CLA	353.0	191.0	−40	−4	−26	−5
CCLA	353.1	191.0	−38	−4	−26	−4
Caffeic acid	179.1	135.1	−33	−4	−22	−7
Rutin	609.3	300.2	−80	−9	−50	−7
Isoquercitrin	463.3	300.1	−57	−8	−35	−8
IAB	515.3	353.2	−45	−6	−20	−3.5
IAA	515.2	353.0	−55	−6	−23	−4
IAC	515.3	352.9	−48	−6	−22	−8
Tussilagone	391.4	217.4	30	4	12.5	3
Ferulic acid (IS1)	192.9	133.8	−28	−6	−21	−4.5
Puerarin (IS2)	415.1	267.2	−60	−8	−45	−6.5
Artemisinin (IS3)	283.3	151.3	20	10	18	3

**Table 2:** Regression equation, linear range, and LLOQs of 10 compounds (*n* = 6).

Compounds	Regression equation	*r* ^2^	Linear range	LLOQ (ng·mL^−1^)
NCLA	*Y* = 0.0109*X* − 0.00382	0.9984	2–6000	2
CLA	*Y* = 0.0649*X* + 0.00231	0.9993	1–3000	1
CCLA	*Y* = 0.00475*X* − 0.00501	0.9972	2–6000	2
Caffeic acid	*Y* = 0.0322*X* − 0.393	0.9937	10–30000	10
Rutin	*Y* = 0.00247*X* + 0.00135	0.9983	1–3000	1
Isoquercitrin	*Y* = 0.00707*X* + 0.00553	0.9983	1–3000	1
IAB	*Y* = 0.00264*X* − 0.00961	0.9952	4–12000	4
IA A	*Y* = 0.00135*X* − 0.00307	0.9917	4–12000	4
IAC	*Y* = 0.0102*X* − 0.0172	0.9934	2–6000	2
Tussilagone	*Y* = 0.00801*X* + 0.00969	0.9964	1–3000	1

**Table 3:** Accuracies and precisions of ten compounds (*n* = 6).

Compounds	Concentration (ng·mL^−1^)	Intraday		Interday	
		Accuracy (%)	RSD (%)	Accuracy (%)	RSD (%)
NCLA	LLOQ	106	13.4	97.1	17.5
	6	92.7	11.2	91.8	10.9
	200	91.2	3.69	101	11.8
	6000	95.7	3.85	99.9	6.24

CLA	LLOQ	95.9	11.7	102	13.8
	3	89.1	6.38	90.0	12.3
	100	111	2.58	103	8.15
	3000	113	4.58	115	9.33

Cryptochlorogenic acid	LLOQ	103	9.21	112	14.2
	6	106	9.67	102	11.1
	200	114	14.2	104	13.7
	6000	109	5.99	99.9	10.4

Caffeic acid	LLOQ	109	2.75	109	13.2
	30	97.1	13.5	102	13.8
	1000	113	5.34	114	10.3
	30000	89.4	5.29	94.3	12.2

Rutin	LLOQ	90.8	8.38	86.3	12.4
	3	114	7.94	113	9.25
	100	87.1	14.3	97.9	13.8
	3000	89.3	12.6	96.8	12.7

Isoquercitrin	LLOQ	87.8	13.2	80.6	13.1
	3	113	6.81	106	11.7
	100	108	3.06	102	8.93
	3000	104	8.69	101	14.2

IAB	LLOQ	81.9	4.90	92.1	13.9
	12	93.9	12.7	89.1	13.8
	400	98.9	6.13	104	7.85
	12000	106	5.10	104	7.68

IAA	LLOQ	110	14.6	99.5	15.2
	12	91.3	14.3	86.2	13.9
	400	106	6.87	107	12.6
	12000	105	2.39	113	7.84

IAC	LLOQ	81.8	14.3	88.5	17.5
	6	88.8	12.8	85.9	11.2
	200	113	6.58	109	10.4
	6000	104	7.54	103	11.5

Tussilagone	LLOQ	88.0	6.72	86.1	11.6
	3	87.3	7.08	97.0	9.39
	100	91.6	13.3	103	13.1
	3000	102	12.3	96.3	12.5

**Table 4:** Recoveries and matrix effects of ten compounds (*n* = 6).

Compounds	Concentration (ng·mL^−1^)	Recovery (%)	RSD (%)	Matrix effect (%)	RSD (%)
NCLA	6	84.2	7.67	113	8.82
	200	80.3	3.24	85.9	5.68
	6000	87.1	6.46	87.5	12.1

CLA	3	92.4	3.99	114	12.3
	100	96.5	3.19	112	14.2
	3000	87.9	6.89	113	5.02

CCLA	6	102	12.2	113	5.96
	200	94.7	9.38	112	6.59
	6000	98.2	6.56	99.6	13.1

Caffeic acid	30	107	11.4	88.5	8.68
	1000	94.4	5.05	101	1.58
	30000	98.5	9.24	87.1	7.59

Rutin	3	78.1	9.38	88.3	9.96
	100	82.6	5.76	85.4	7.80
	3000	84.7	14.2	110	5.62

Isoquercitrin	3	103	7.39	90.7	10.1
	100	101	4.95	86.9	7.61
	3000	93.0	6.22	89.5	12.8

IAB	12	85.9	10.6	113	9.76
	400	102	3.16	114	5.23
	12000	95.6	10.0	111	9.01

IAA	12	90.6	10.0	85.2	8.51
	400	94.2	6.60	85.1	8.64
	12000	94.5	6.07	113	10.1

IAC	2	91.8	14.8	105	10.6
	200	97.1	13.2	110	10.0
	6000	98.5	2.94	113	8.22

Tussilagone	3	96.6	12.0	91.0	5.41
	100	114	3.28	86.5	6.81
	3000	91.0	13.0	86.9	7.95
IS_1_	100	74.0	5.34	95.6	1.86
IS_2_	100	70.3	5.00	95.6	11.0
IS_3_	100	70.9	9.16	91.9	2.80

**Table 5:** Stability of 10 compounds.

Compounds	Concentration (ng·mL^−1^)	24 h stability		Free-throw stability		Long stability	
		Remain (%)	RSD (%)	Remain (%)	RSD (%)	Remain (%)	RSD (%)
NCLA	6	93.5	13.8	111	5.39	107	6.22
	200	103	7.36	82.9	2.78	108	14.8
	6000	92.4	4.59	87.0	3.39	114	10.5

CLA	3	99.4	13.7	103	13.2	114	6.31
	100	103	9.71	88.7	4.09	93.6	13.2
	3000	101	4.33	115	4.05	112	12.3

CCLA	6	98.4	9.83	96.8	4.58	97.5	7.69
	200	112	9.24	82.9	17.4	98.6	12.8
	6000	112	1.63	93.2	3.24	95.9	13.7

Caffeic acid	30	81.1	12.1	101	9.23	113	8.19
	1000	113	3.24	87.2	3.52	109	6.34
	30000	90.7	5.53	85.5	11.6	81.1	3.64

Rutin	3	95.1	11.9	112	7.33	97.0	13.4
	100	109	3.74	114	1.08	99.5	13.4
	3000	107	9.04	116	4.23	89.6	14.8

Isoquercitrin	3	84.4	14.8	104	9.63	92.0	11.6
	100	113	4.76	111	8.68	105	4.56
	3000	113	3.37	110	5.15	92.3	15.0

IAB	12	102	12.5	96.8	11.7	106	11.9
	400	83.7	10.1	94.9	3.06	108	6.57
	12000	94.9	4.09	90.8	2.51	113	5.19

IAA	12	94.5	14.4	97.6	8.38	101	8.71
	400	101	9.82	87.6	1.23	97.6	13.4
	12000	106	4.17	98.5	7.82	107	9.36

IAC	6	91.7	8.79	106	4.35	102	10.0
	200	105	6.37	98.6	8.29	108	13.6
	6000	97.6	3.71	93.7	5.24	106	6.14

Tussilagone	3	100	10.8	83.9	14.5	101	11.4
	100	92.1	13.2	115	14.5	107	12.6
	3000	95.0	11.1	91.8	7.13	109	14.3

**Table 6:** Pharmacokinetic parameters of 10 compounds.

Compounds	Dosage	*T*_max_ (h)	*C*_max_ (ng/mL)	AUC_(0–24h_) (ng/mL *∗* h)	AUC_(0–∞)_ (ng/mL *∗* h)	MRT_(0–24h)_ (h)	MRT_(0–∞)_ (h)	*t*_1/2_ (h)
NCLA	3.9 g·kg^−1^	0.69 ± 0.19	73.98 ± 52.03	210.2 ± 126.4	239.3 ± 125.3	4.35 ± 2.74	7.90 ± 7.34	1.61 ± 1.10
	7.8 g·kg^−1^	0.36 ± 0.13	485.7 ± 272.8	965.7 ± 295.9	1028 ± 3007	6.33 ± 2.33	8.61 ± 2.66	1.49 ± 0.54

CLA	3.9 g·kg^−1^	0.64 ± 0.244	537.9 ± 489.2	1425 ± 1046	1467 ± 1044	4.4 ± 1.71	5.41 ± 2.04	2.16 ± 1.46
	7.8 g·kg^−1^	0.38 ± 0.14	2020 ± 42	3611 ± 1023	3692 ± 1052	4.63 ± 1.36	5.32 ± 1.55	1.28 ± 0.56

CCLA	3.9 g·kg^−1^	0.64 ± 0.20	176.7 ± 131.2	422.3 ± 210.5	585.7 ± 414.0	4.17 ± 1.73	5.16 ± 2.01	1.03 ± 0.53
	7.8 g·kg^−1^	0.39 ± 0.20	1070 ± 739	1938 ± 1401	2019 ± 1397	5.02 ± 2.07	6.23 ± 2.10	1.06 ± 0.58

Caffeic acid	3.9 g·kg^−1^	0.23 ± 0.04	96.26 ± 36.57	338.9 ± 246.4	691.7 ± 477.5	6.47 ± 3.05	14.78 ± 8.46	4.65 ± 4.89
	7.8 g·kg^−1^	0.21 ± 0.04	106.0 ± 24.38	473.5 ± 273.8	723.9 ± 468.5	4.81 ± 3.64	15.60 ± 10.49	2.75 ± 2.71

Rutin	3.9 g kg^−1^	0.39 ± 0.16	17.92 ± 17.60	39.49 ± 59.42	42.77 ± 60.06	1.95 ± 1.06	3.06 ± 1.30	0.61 ± 0.46
	7.8 g·kg^−1^	0.32 ± 0.12	76.66 ± 55.47	104.04 ± 53.04	119.849.6	2.79 ± 0.24	6.02 ± 4.70	0.82 ± 0.51

Isoquercitrin	3.9 g·kg^−1^	0.44 ± 0.26	5.806 ± 5.720	7.284 ± 5.643	9.5592 ± 5.91	3.16 ± 3.46	7.56 ± 6.22	0.88 ± 1.01
	7.8 g·kg^−1^	0.27 ± 0.10	10.22 ± 6.83	16.15 ± 12.24	25.18 ± 13.92	2.81 ± 1.79	9.94 ± 10.42	1.19 ± 1.65

IAB	3.9 g·kg^−1^	0.40 ± 0.26	128.7 ± 197.1	249.5 ± 215.5	366.9 ± 261.1	4.43 ± 2.31	13.99 ± 7.86	1.95 ± 1.43
	7.8 g kg^−1^	0.28 ± 0.12	437.7 ± 342.5	899.9 ± 304.5	1175 ± 339	7.22 ± 2.16	14.67 ± 3.61	1.06 ± 0.69

IAA	3.9 g·kg^−1^	0.35 ± 0.14	357.4 ± 333.0	788.6 ± 635.91	947.6 ± 658.00	5.81 ± 2.79	10.02 ± 3.40	1.49 ± 1.20
	7.8 g·kg^−1^	0.28 ± 0.11	1428 ± 437.	2424 ± 1079	25934 ± 1124	6.52 ± 2.17	9.14 ± 2.43	1.16 ± 0.59

IAC	3.9 g·kg^−1^	0.56 ± 0.25	47.27 ± 29.65	187 ± 85.5	319.1 ± 217.3	5.79 ± 3.25	18.7 ± 18.60	4.25 ± 6.16
	7.8 g·kg^−1^	0.35 ± 0.21	264.57 ± 95.55	823.4 ± 182.9	941.9 ± 204.7	8.80 ± 2.73	13.78 ± 4.86	1.37 ± 0.83

Tussilagone	3.9 g·kg^−1^	0.44 ± 0.38	0.83 ± 0.50	1.06 ± 0.564	2.19 ± 1.98	0.87 ± 0.134	3.12 ± 2.62	0.28 ± 0.20
	7.8 g·kg^−1^	0.40 ± 0.33	18.07 ± 13.00	35.48 ± 42.302	47.12 ± 49.445	2.42 ± 2.10	6.98 ± 9.13	0.82 ± 0.72

## Data Availability

The data used to support the findings of this study are included in this article.
